# Preparation and Characterization of Paraffin/Mesoporous Silica Shape-Stabilized Phase Change Materials for Building Thermal Insulation

**DOI:** 10.3390/ma14071775

**Published:** 2021-04-03

**Authors:** Yong Li, Mingyue Dong, Wang Song, Xiaoyu Liang, Yaowen Chen, Yanfeng Liu

**Affiliations:** 1 State Key Laboratory of Green Building in Western China, Xi’an University of Architecture and Technology, No. 13 Yanta Road, Xi’an 710055, China; yanfengliu@xauat.edu.cn; 2 School of Building Services Science and Engineering, Xi’an University of Architecture and Technology, No. 13 Yanta Road, Xi’an 710055, China; dmy@xauat.edu.cn (M.D.); songwangaa66@163.com (W.S.); xiaoyuliang2021@163.com (X.L.); yaowen@xauat.edu.cn (Y.C.)

**Keywords:** phase change material, mesoporous silica, shape-stabilized, thermal storage, thermal insulation

## Abstract

The use of phase change materials (PCMs) is an attractive method for energy storage and utilization in building envelopes. Here, shape-stabilized phase change materials (SS-PCMs) were prepared via direct adsorption using mesoporous silica (MS) with different pore diameters as the support matrix. The leakage properties, microstructure, chemical structure, thermophysical properties, activation energy, thermal stability and thermal storage-release characteristics of paraffin and SS-PCMs were investigated. The results show that the maximum mass proportion of paraffin in SS-PCMs is 70% when the average pore diameter of mesoporous silica is 15 nm, and the phase change temperature and latent heat of the corresponding SS-PCM are 23.6 °C and 135.4 kJ/kg, respectively. No chemical reaction occurs between mesoporous silica and paraffin and the SS-PCMs exhibit high thermal stability. The high activation energy of the paraffin (70%)/MS1 SS-PCM verifies that the shape and thermal properties can be maintained stably during phase change conversions. The time required for SS-PCMs to complete the thermal storage and release process is reduced by up to 34.0% compared with that for pure paraffin, showing a decline in the thermal conductivity of SS-PCMs after the addition of mesoporous silica. Hence, the prepared paraffin/MS SS-PCMs, in particular paraffin (70%)/MS1 SS-PCM, can be used for storing thermal energy and regulating indoor temperature in buildings.

## 1. Introduction

With the rising demand for building thermal comfort and high-quality indoor thermal environment, building energy consumption has been growing continuously in recent years [1,2,3,4]. Thus, various environment-friendly technologies have been proposed to enhance building energy efficiency and reduce carbon dioxide emissions [5,6]. Among various technologies, the use of phase change materials (PCMs) has gained more and more attention, as they have high energy storage density and can be operated within a small temperature range during the phase change conversion process [7,8,9]. However, PCMs are prone to leakage after melting when they are directly incorporated into building structures, which limits their wide application in building energy conservation [10,11].

In order to solve the leakage problem of the melted PCMs, composite PCMs are prepared by micro-encapsulation methods, sol-gel methods, pressing sintering methods, direct adsorption methods, etc. [12,13,14]. In particular, shape-stabilized phase change materials (SS-PCMs) consisting of one or several types of PCMs and a supporting porous matrix, such as graphite [15], carbon foam [16], or carbon nanotubes [17], are generally preferred. The investigations on PCMs mainly focus on the improvement of thermal conductivity [18]. However, good thermal insulation and thermal mass are both essential to improve indoor thermal comfort and reduce building energy consumption. Thus, SS-PCMs with low thermal conductivity are preferred for application in buildings [19]. In order to decrease the thermal conductivity of PCMs, one of the most efficient approaches is adding porous matrices with low thermal conductivity to prepared SS-PCMs. For example, Lv et al. [20] prepared paraffin/kaolin composites by employing kaolin with different particle sizes and studied the thermal performance of pure PCM and PCM composites. Zhang et al. [21] prepared a PCM composite consisting of a MgCl_2_·6H_2_O-based eutectic and expanded perlite. The maximum mass fraction of eutectic hydrated salt in the composite was 50 wt.%, and the thermal conductivity of the PCM was reduced from 0.732 to 0.144 W/(m·K). Sun et al. [22] prepared an SS-PCM by combining binary mixtures of CaCl_2_·6H_2_O-NH_4_Cl with expanded perlite and found that the thermal conductivity of PCM was reduced to 0.117 W/(m·K).

Mesoporous silica (MS), which has the characteristics of low thermal conductivity, large specific surface area, ultralight weight and outstanding compatibility with construction materials, is considered as a preferable supporting matrix for the preparation of SS-PCMs [23]. Luo et al. [19] prepared an SS-PCM by mixing a ternary eutectic salt with MS. The latent heat and thermal conductivity of the SS-PCM were 99.43 kJ/kg and 0.082 W/(m·K), respectively. Luo et al. [24] prepared a novel paraffin@SiO_2_ SS-PCM by a chemical precipitation process, and the thermal conductivity of the composite was significantly improved compared with pure PCM. Ranjbar et al. [25] prepared a PCM nanocomposite based on a *n*-heptadecane core and SiO_2_ nanoparticles framework. The results showed that the nanocomposite had an enthalpy of 123.8 J/g and a good thermal reliability. Li et al. [26] integrated the paraffin/MS composites into vacuum insulation panels and found that the thermal mass of the vacuum insulation panels was increased with integration of PCM. Nomura et al. [27] developed SS-PCMs for building materials by vacuum impregnation of octadecane into MS, with SS-PCM impregnation ratio reaching 0.95. 

Although some studies have been conducted on the application of SS-PCMs consisting of organic PCM and MS, there is very little data regarding the influence of pore diameters of MS on the insulation properties of paraffin/MS SS-PCMs. Herein, paraffin/MS SS-PCMs were prepared by direct adsorption method employing MS with average particle diameters of 15, 30 and 50 nm, respectively. Furthermore, the chemical characteristics, microstructure, thermophysical properties, activation energy and thermal stability were systematically studied by FTIR, SEM, DSC and TGA, respectively. Finally, the thermal storage/release performances of paraffin and SS-PCMs were investigated by measuring the temperature evolution during the heat charging and discharging process.

## 2. Materials and Methods

### 2.1. Materials

PCM: paraffin was provided by Joule Wax Industry Co., Ltd. (Shanghai, China), and its phase change temperature was 28 °C. Amorphous mesoporous silica was supplied by Shanghai Macklin Biochemical Technology Co., Ltd. (Shanghai, China). The average pore sizes of mesoporous silica were 15 nm, 30 nm and 50 nm, respectively, represented by MS1, MS2 and MS3. The average specific surface areas for MS1, MS2 and MS3 were 230 m^2^/g, 180 m^2^/g and 150 m^2^/g, with an up and down fluctuation of 30 m^2^/g. The pH value of MS was in the range of 5–7, the loss on ignition at 1000 °C was no more than 2% and the purity of MS was higher than 99.5%. 

### 2.2. Preparation

A direct adsorption method was used to prepare the SS-PCMs. A certain quantity of paraffin was weighed and placed in a beaker, which was heated in a constant temperature drying box at 50 °C. After the paraffin was completely melted, it was added into mesoporous silica with different pore sizes. To ensure that the paraffin was evenly distributed in the mesoporous silica, the beaker containing PCM composites was placed in a hot water bath at a temperature of 50 °C and stirred for 30 min at 300 r/m. After that, the PCM composites were cooled in a refrigerator at 4 °C. A series of SS-PCM samples with different mass percentages of paraffin was prepared, and the mass ratios of paraffin and mesoporous silica in SS-PCMs are listed in Table 1.

### 2.3. Characterization

Leakage tests were carried out to determine the maximum mass proportion of paraffin in SS-PCMs. Filter papers with SS-PCM samples were placed in a constant temperature drying box at 50 °C for 1 h to ensure that the phase change material melted completely. After that, the filter paper was taken out to observe the leakage trace to determine whether the PCM leaked from the SS-PCMs. 

The chemical structures of mesoporous silica, paraffin and SS-PCMs were analyzed by Fourier transform infrared spectrometer (FTIR, Nicolet IS50, Thermo Scientific, Waltham, MA, USA) after the blank potassium bromide plate and sample were pressed. The wavenumber varied from 400 to 4000 cm^−1^. The microstructures of mesoporous silica and SS-PCM samples were observed by scanning electron microscope (SEM, Geminisem 500, Carl Zeiss, Jena, Germany). The PCM samples were sprayed with gold and tested at an ambient temperature of 20 °C. The thermal properties were verified by differential scanning calorimeter (DSC, 214 Polyma, Netzsch, Selb, Germany) in a nitrogen environment. The temperature was in the range of 5–50 °C and the heating rate was 5 °C/min. The heat flow and temperature accuracy of DSC were ±1% and ±0.1 °C, respectively. DSC tests for SS-PCMs were carried out to analyze the activation energy under different heating rates of 2 °C /min, 5 °C /min, 10 °C /min, 15 °C /min, respectively. A thermo-gravimetric analyzer (TGA, STA449C, Netzsch) was used to study the thermal stability of the samples under nitrogen protection. The temperature was varied from room temperature (25 °C) to 600 °C and the heating rate was 10 °C/min.

**Figure 1:**
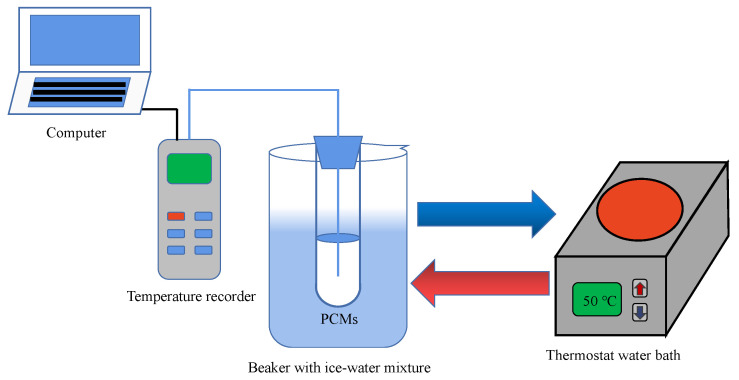
Schematic of the thermal performance evaluation system of paraffin and SS-PCMs.

Thermal storage/release performances of paraffin and SS-PCMs were studied by measuring the temperature evolution of the PCMs. The schematic of the thermal performance evaluation system is shown in Figure 1. The PCM sample with a weight of 15 g was packed into a test tube and then placed in a beaker containing ice-water mixture for 20 min. After that, the test tube was taken out and placed in a constant temperature water bath at 50 °C for testing the heat storage performance of PCMs. After the temperature of PCMs reached a stable value, the test tube was taken out of the water bath and placed into the beaker containing ice-water mixture to start the heat release process of PCMs. A T type thermocouple device was used to measure the temperature curves of the SS-PCMs. The accuracy of the thermocouple was ±0.5 °C, and the data were collected every 30 s by a data acquisition system (34970A, Agilent, Santa Clara, CA, USA). 

## 3. Results and Discussion

### 3.1. Leakage Performance 

**Figure 2:**
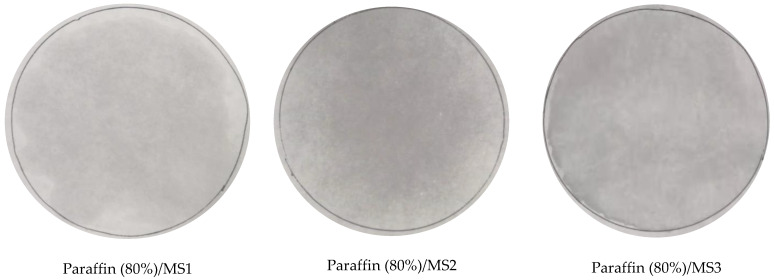

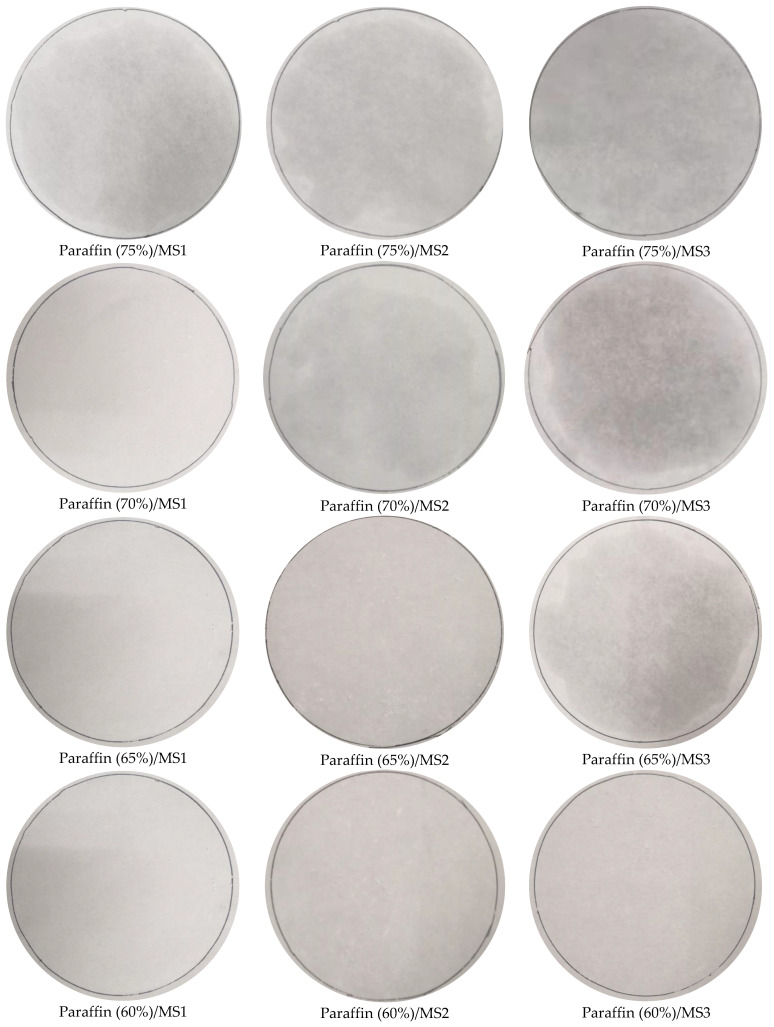

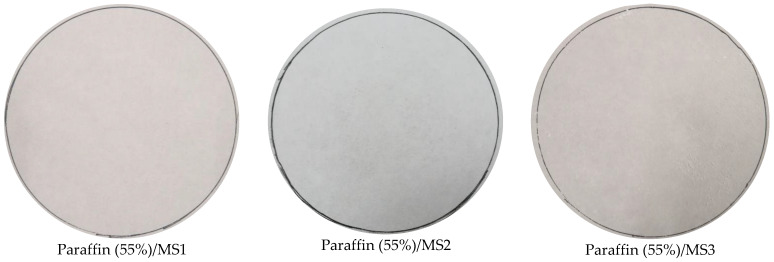
Leakage test of MS1, MS2 and MS3 SS-PCMs with different paraffin mass proportion after heating.

Leakage of the melted PCM is considered a serious problem when a PCM is used in buildings, thus the maximum absorption proportion of PCM in SS-PCMs is determined by the leakage test. Figure 2 shows the leakage test results of MS1, MS2 and MS3 SS-PCMs with different paraffin mass fractions after heating. As seen in the Figure 2, when the mass fraction of paraffin in SS-PCMs is 80%, the leakage trace was observed for the paraffin (80%)/MS1, paraffin (80%)/MS2 and paraffin (80%)/MS3 SS-PCMs because it has exceeded the maximum absorption capability of MS. Some leakage is visible for the paraffin (80%)/MS1, while serious leakage occurs for paraffin (80%)/MS3. As for paraffin (75%)/MS1 SS-PCMs, a little leakage can be observed from the leakage traces. When the mass fraction of paraffin is 70% or less in paraffin/MS1 SS-PCM, no traces of leakage can be found on the filter paper. This indicates that mesoporous silica is an effective supporting substrate for the melted paraffin when the mass proportion of paraffin is less than or equal to 70%, thus enhancing the stability of the paraffin/MS1 SS-PCM. The same procedure was repeated for paraffin/MS2 and paraffin/MS3 SS-PCM. For MS2 and MS3, the maximum mass proportions of paraffin corresponding to no leakage are 65% and 60%, respectively. Therefore, the PCMs used in subsequent studies are paraffin (70%)/MS1, paraffin (65%)/MS2, and paraffin (60%)/MS3 SS-PCMs.

### 3.2. Chemical Characteristics 

**Figure 3:**
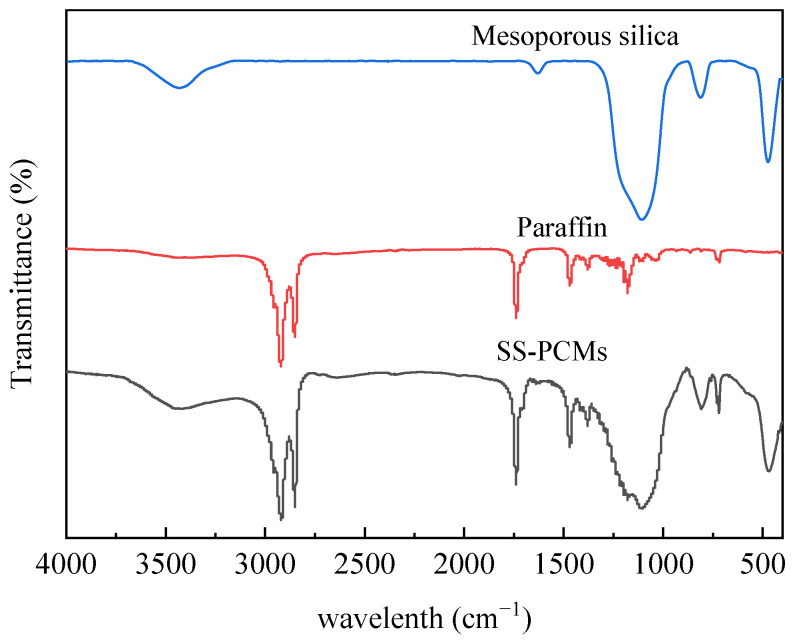
FT-IR spectra of paraffin, MS and paraffin/MS SS-PCMs.

The chemical structure analysis of the PCMs and MS was performed by FTIR. The FTIR spectra of mesoporous silica, paraffin and paraffin/mesoporous silica are exhibited in Figure 3. In the FTIR spectrum of paraffin, the absorption peaks appeared at 2920 and 2851 cm^−1^ are assigned to -CH_2_ and -CH_3_ stretching vibrations, while the absorption peak at 1744 cm^−1^ is assigned to C=O stretching vibration in paraffin. The absorption peaks at 1468 and 1179 cm^−1^ are assigned to -CH_2_ and -CH_3_ in-plane bending vibrations, respectively, and the absorption peak at 718 cm^−1^ corresponds to -CH_2_ in-plane swing vibration. In the FTIR spectrum of SiO_2_, a wide band caused by Si-OH stretching vibration appears at 3000–3600 cm^−1^. The bands at 472.54 cm^−1^, 812.01 cm^−1^ and 1107.11 cm^−1^ correspond to bending vibrations of Si-O-Si, symmetric stretching vibrations and asymmetric stretching vibrations, respectively. Paraffin/MS SS-PCMs have all the characteristic absorption peaks of paraffin and mesoporous silica, and no new peaks of other functional groups are generated. This verifies that there is no chemical reaction between paraffin and mesoporous silica, and there is only a slight shift of the characteristic peaks due to the physical reaction. A jagged FTIR spectrum of paraffin and SS-PCM is observed over the wavenumber range of 1200 to 1375 cm^−1^. This is probably because raw paraffin was employed in the present study and the impurities in paraffin result in the jaggedness of the FTIR spectra [28,29]. 

### 3.3. Material Morphology

**Figure 4:**
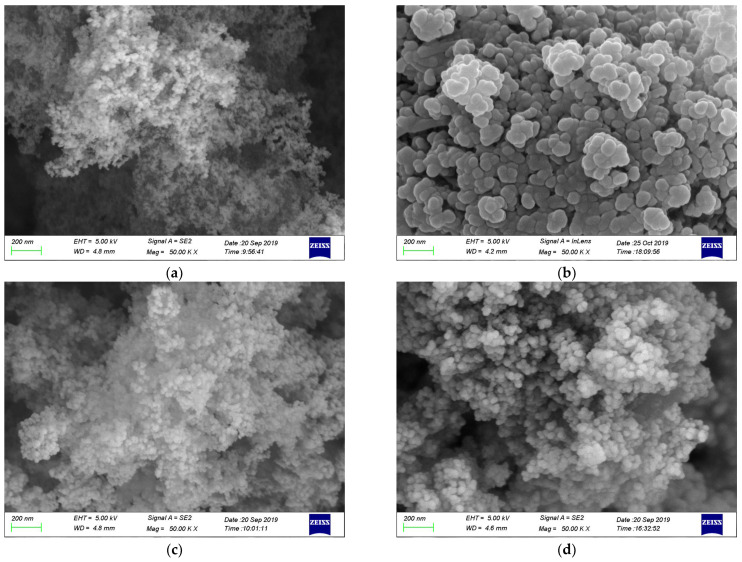

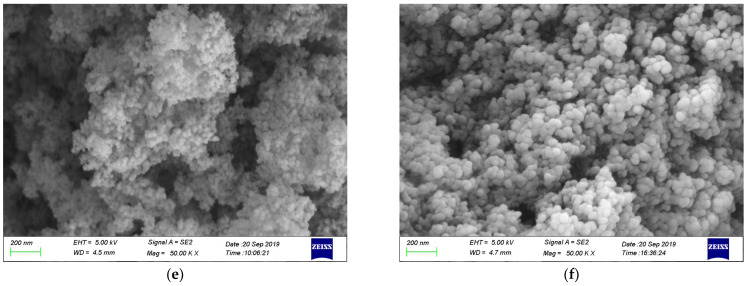
SEM images of mesoporous silica and paraffin/MS SS-PCMs:(**a**) MS1; (**b**)Paraffin (70%)/MS1; (**c**) MS2; (**d**) Paraffin (65%)/MS2; (**e**) MS3; (**f**) Paraffin (60%)/MS3.

The microstructure and morphology of mesoporous silica and paraffin/MS SS-PCMs are shown in Figure 4. It can be observed from Figure 4a,c,d that mesoporous silica samples with different particle diameters all have highly porous structures between the particles. These porous structures are responsible for the good adsorption performance of mesoporous silica. Thus, the leakage problem of the PCM in liquid state can be effectively avoided by the surface tension and the capillary action of mesoporous silica, which can explain the leakage performance of SS-PCMs visually [30]. As exhibited in Figure 4b,d,f, when the mass fractions of paraffin in SS-PCMs are 70%, 65% and 60%, the pore structures of mesoporous silica are filled with paraffin, and the surface of mesoporous silica becomes round and smooth. The surface smoothness of paraffin/MS SS-PCMs with the pore diameter of 15 nm is the best, as the surface area of mesoporous silica increases with the decrease in particle size [7].

**Figure 5:**
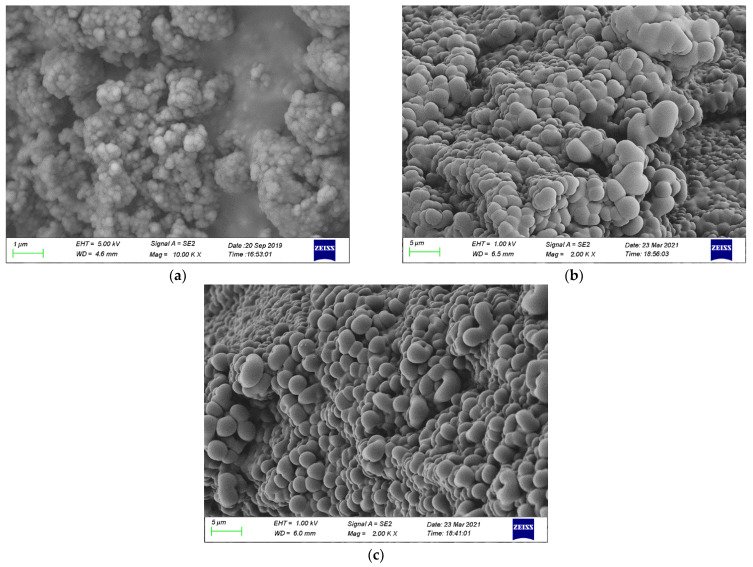
SEM images of paraffin (70%)/MS SS-PCMs with different particle size of MS:(**a**) paraffin (70%)/MS1 SS-PCM; (**b**) paraffin (70%)/MS2 SS-PCM; (**c**) paraffin (70%)/MS3 SS-PCMs.

The SEM images of paraffin (70%)/MS SS-PCMs with different particle size of MS are provided for comparison, as shown in Figure 5. It can be seen that paraffin does not completely fill the porous structure of MS for paraffin (70%)/MS1 SS-PCM. With the increasing particle size of MS, the surface of SS-PCM turns smooth and round, as shown in Figure 5b,c. However, the MS nanoparticles and the porous structure between the particles cannot supply enough surface tension and capillary force to prevent melted paraffin leaking from the porous structure of silica. Thus, leakage occurs for paraffin (70%)/MS2 SS-PCM and paraffin (70%)/MS3 SS-PCM as the mass fraction of paraffin in SS-PCM exceeds its maximum absorption ability, which has been verified by the leakage tests.

### 3.4. Thermophysical Properties of SS-PCMs

**Figure 6:**
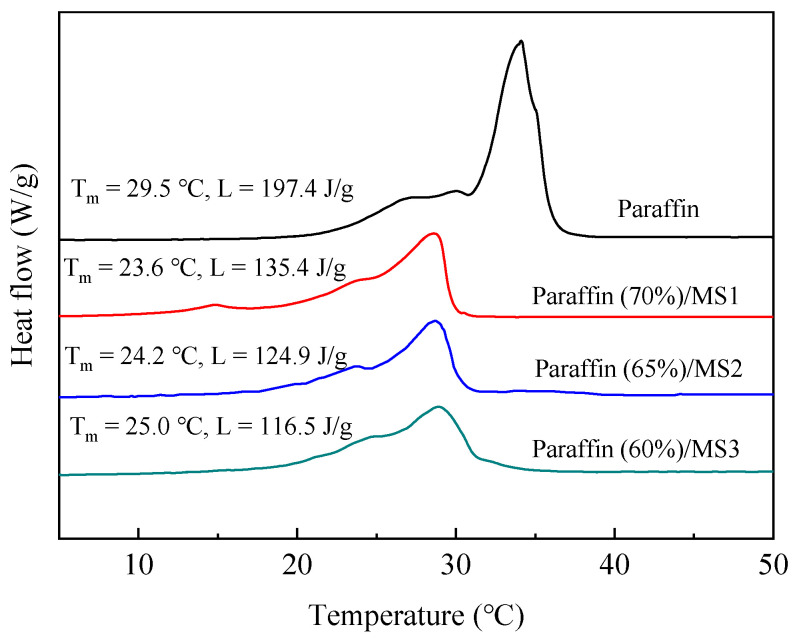
DSC curves of paraffin and paraffin/MS SS-PCMs with different MS pore diameters and maximum paraffin mass proportions of 70%, 65% and 60%.

The DSC thermogram of paraffin and paraffin/MS SS-PCMs containing mesoporous silica with different particle diameters are shown in Figure 6. In the SS-PCMs, only paraffin can store and release latent heat during the phase change conversion process. Thus, the latent heat of SS-PCMs with different particle diameters of MS is determined by the mass proportion of paraffin. The theoretical value of the latent heat of SS-PCMs is calculated by the following equation [31,32]:

*L*_theoretical_ = *L*_paraffin_ × *η*% (1)

where L_theoretical_ is the theoretical value of the latent heat of the SS-PCMs, L_paraffin_ represents the latent heat of paraffin and η represents the mass proportion of paraffin in SS-PCMs.

The crystallization efficiency of paraffin within the SS-PCMs can be expressed as follows [33,34]:

*α = [L_test_/(L_paraffin_× η)]* × 100% (2)

where *α* is the crystallization of paraffin in SS-PCMs, and *L_test_* is the latent heat value of SS-PCMs obtained by DSC.

The theoretical and measured values of the latent heat of the SS-PCMs and the crystallization efficiency of paraffin in the SS-PCMs are shown in Table 2. The latent heats of paraffin (70%)/MS1, paraffin (65%)/MS2 and paraffin (60%)/MS3 are 135.4 kJ/kg, 124.9 kJ/kg and 116.5 kJ/kg, respectively. The molecular movement of paraffin is restricted due to the mesoporous structure of silica. Therefore, a small proportion of the paraffin cannot melt or solidify [35]. Consequently, the measured latent heat value is lower than the theoretical value. The phase change temperature of SS-PCMs with different pore diameters is reduced by 4.5–5.9 °C compared to that of pure paraffin. This is probably caused by the weak interaction between the paraffin molecules and the surface of silica [30]. All the crystallization values of paraffin within paraffin/MS SS-PCMs are close to 100%. This result verifies that paraffin in SS-PCM can undergo crystallization and phase change conversions with reversible efficiency. 

### 3.5. Activation Energies Analysis

The activation energy is equal to the height of the activation barrier, which is used to describe the relationship between the reaction rate and temperature. The activation energy is calculated by Kissinger method [36,37], which follows the kinetic equation:

(3) $$\ln\left(\frac{\beta }{T_{p}^{2}}\right)=-\frac{E_{C}}{RT_{p}}+C$$

where β represents the heating rate (K/min), $T_{p}$ represents the melting peak temperature (°C), and $E_{C}$ represents the activation energy of PCM (kJ/mol). 

**Figure 7:**
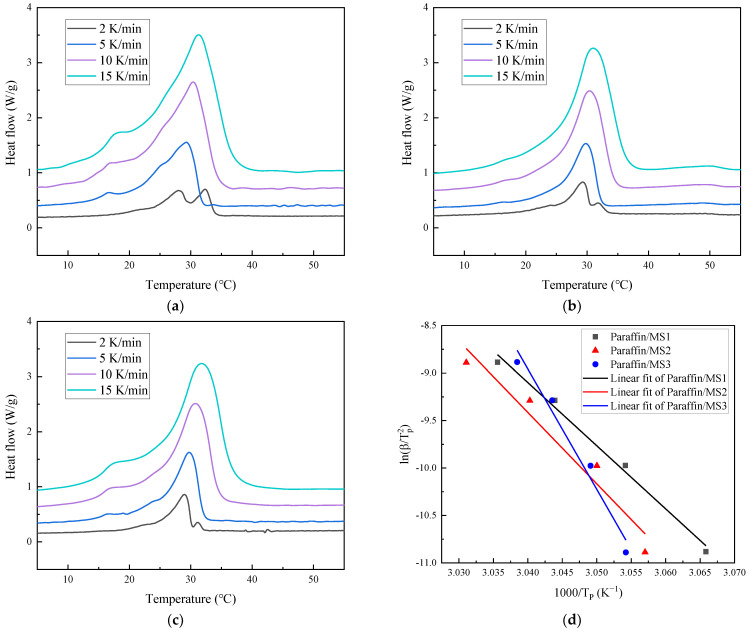
Data for activation energy calculations: (**a**) DSC results for paraffin/MS1 SS-PCM at different heating rates, (**b**) DSC results for paraffin/MS2 SS-PCM at different heating rates, (**c**) DSC results for paraffin/MS3 SS-PCM at different heating rates, (**d**) the linear fitting of $\ln\left(\frac{\beta }{T_{p}^{2}}\right)$ vs. $\frac{1000}{T_{p}}$ based on the Kissinger equation.

DSC tests for paraffin and SS-PCM were carried out under different heating rates of 2 °C /min, 5 °C /min, 10 °C /min, 15 °C /min, respectively. With the increase in heating rate, the endothermic peak of PCM is wider and the peak temperature is higher. Moreover, the phase change temperature of PCM moves to a higher value. This is mainly because the higher the heating rate, the more obvious the hysteresis of the heat transfer process. Using the Kissinger dynamic method, the linear fitting of $\ln\left(\frac{\beta }{T_{p}^{2}}\right)$ vs. $\frac{1000}{T_{p}}$ is obtained, as shown in Figure 7. The activation energies of the SS-PCMs under the four different heating rates are shown in Table 3. The fitting correlation coefficients are all higher than 0.94, which indicates that a good relationship between $\ln\left(\frac{\beta }{T_{p}^{2}}\right)$ and $\frac{1000}{T_{p}}$ can be obtained. The activation energy values of paraffin (70%)/MS1 SS-PCM, paraffin (65%)/MS2 SS-PCM, paraffin (60%)/MS3 SS-PCM are calculated as 1053.72 kJ/mol, 625.38 kJ/mol and 553.29 kJ/mol, respectively. From the comparison, it can be seen that paraffin (70%)/MS1 SS-PCM has a larger activation energy value. That is probably because that the restriction on the movement of paraffin molecule increases as the pore size of the mesoporous silica decreases. On the other hand, the high activation energy of the paraffin (70%)/MS1 SS-PCM verifies that the shape and thermal properties can be maintained stably during phase change conversion in thermal storage applications [37]. 

### 3.6. Thermal Stability

**Figure 8:**
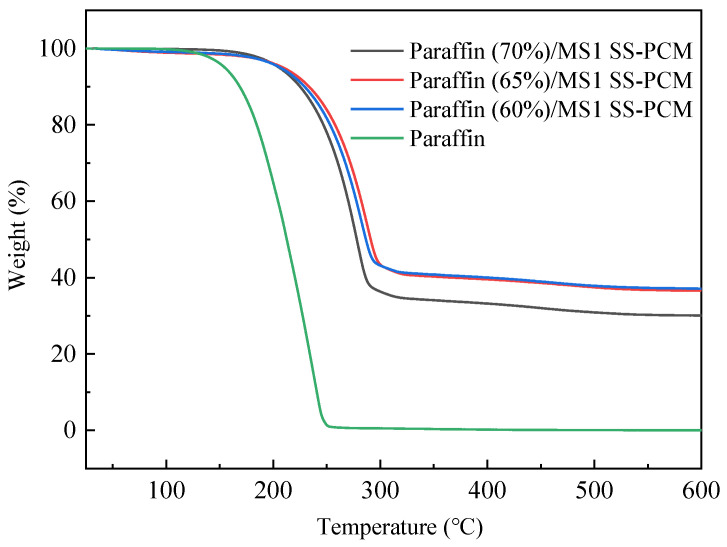
TGA curves of paraffin and paraffin/MS SS-PCMs containing MS with different pore diameters.

Figure 8 illustrates the TGA curves of PCMs containing MS with different pore diameters. It can be seen that there is almost no paraffin weight loss when the temperature is lower than 100 °C. The weight loss starting point is pushed back after the addition of mesoporous silica into paraffin, as a result of the physical barrier of mesoporous silica structure in three dimensions. As the temperature reaches up to 250 °C, the weight loss of paraffin is almost complete, whereas the weight loss of paraffin/MS SS-PCMs occurs at temperature above 300 °C, demonstrating that the thermal stability of the paraffin/MS SS-PCMs is enhanced after the addition of mesoporous silica. The weight loss values of paraffin/MS1, paraffin/MS2 and paraffin/MS3 SS-PCMs are 69.9%, 63.4% and 62.9%, respectively. These data are approximately equal to the theoretical mass proportions of paraffin inside SS-PCMs, except for paraffin/MS3 SS-PCMs, which is a bit higher than its theoretical value. One possible reason is that a very small mass of the sample is used for the weight loss measurement, and the mass proportion of paraffin deviates to the expected value (60%). For paraffin/MS1, paraffin/MS2 and paraffin/MS3 SS-PCMs, the weight loss values are 4.1%, 3.9% and 4.1%, respectively, when the temperature is below 200 °C, while the weight loss of pure paraffin is 35.1%. These results indicate that the paraffin/MS SS-PCMs have good thermal stability for the intended building material application.

### 3.7. Thermal Energy Performance of SS-PCMs and Paraffin

**Figure 9:**
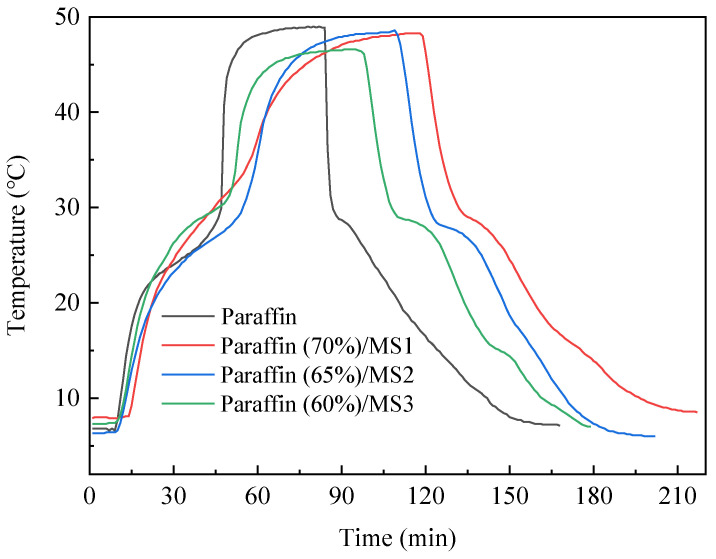
Temperature evolution of paraffin and paraffin/MS SS-PCMs with different MS pore diameters during the heat charging and discharging process.

The thermal energy storage/release performance of paraffin and SS-PCMs was studied experimentally, as shown in Figure 9. The thermal storage and release rate of pure PCM is higher than that of SS-PCMs with different pore diameters, indicating that thermal conductivity of PCM decreases after the addition of mesoporous silica. It takes about 80 min for pure PCM to finish the heat charging process, while the three kinds of SS-PCMs take about 118 min (paraffin/MS1), 104 min (paraffin/MS2) and 92 min (paraffin/MS3), respectively, showing a maximum increase of 47.5%. The time required for paraffin to complete the total heat storage and release process is 162 min, while the corresponding times for paraffin/MS1, paraffin/MS2 and paraffin/MS3 SS-PCMs are 217 min, 198 min and 179 min, respectively, showing a maximum increase of 34.0% for paraffin/MS1. By comparing the heat charging and discharging times of paraffin and SS-PCMs, it is obvious that the thermal storage and release rates of PCM are greatly decreased after adding mesoporous silica. Theoretically, the thermal conductivity of mesoporous silica decreases with decrease in the pore diameter [21]. Hence, SS-PCM with smaller pore diameter exhibits slower thermal storage and release rate. As a result, it takes a longer time for paraffin/MS1 composite to finish the heat storage and release process compared to paraffin and other PCM composites. These results indicate that the SS-PCMs in the present study are suitable for storing thermal energy and regulating indoor temperature in buildings.

### 3.8. Discussion

In this section, the main findings of the present study are compared with those of some SS-PCMs in literature. Table 4 displays a comparison of the thermophysical properties of paraffin/MS SS-PCMs with those of some SS-PCMs in literature. It can be seen that the mesoporous silica in the present study has relatively high absorption ratio in the range of 60–70% and latent heat capability in the range of 116.5–135.4 J/g. 

The addition of porous material with low thermal conductivity can improve the thermal insulation property of PCM. Rao et al. [40] prepared SS-PCM composed of eutectic hydrated salt as PCM and expanded perlite as supporting matrix. They found that the heat storage time of PCM composite insulation panel was extended by 79%, indicating a better thermal insulation performance compared to the pure expanded perlite panel. Qu et al. [41] reported that the heat storage time of concrete block was extended by 62.1% when fumed silica PCM composite with a mass proportion of 20% was added. In the present study, the times required for SS-PCMs to complete the heat storage process and the overall heat charging and discharging process are reduced by up to 47.5% and 34.0%, respectively, compared with that for pure paraffin. This indicates that the paraffin/MS SS-PCM in the present study exhibits good thermal insulation property by the addition of mesoporous silica with low thermal conductivity.

From the above analysis, it can be seen that the maximum mass proportion of paraffin inside SS-PCM is 70% when the pore diameter of mesoporous silica is 15 nm, which is higher than that of the other two SS-PCMs with the pore diameter of 30 nm and 50 nm, respectively. Due to the high absorption proportion of paraffin, paraffin (70%)/MS1 SS-PCM exhibits a latent heat of 135.4 kJ/kg and phase change temperature of 23.6 °C. It also takes a longer time for paraffin (70%)/MS1 SS-PCM to complete the heat charging and discharging process, showing a better thermal insulation property compared to paraffin (65%)/MS2 and paraffin (60%)/MS3 SS-PCMs. Thus, it can be concluded that the paraffin (70%)/MS1 SS-PCM in the present study has high potential for practical application in building envelopes.

## 4. Conclusions

In this work, shape stabilized phase change materials (SS-PCMs) were prepared via a direct adsorption approach using mesoporous silica with three different pore diameters as the support matrix. The average pore sizes of mesoporous silica were 15, 30 and 50 nm, respectively, represented by MS1, MS2 and MS3. The leakage property, microstructure, chemical structure, thermophysical properties, thermal stability and thermal performance of paraffin and the SS-PCMs were characterized. The integration of mesoporous silica into paraffin leads to the reduction in phase change temperature and latent heat. The maximum mass proportion of paraffin inside SS-PCMs is 70% when the pore diameter of mesoporous silica is 15 nm, and the SS-PCM exhibits a phase change temperature of 23.6 °C and latent heat of 135.4 kJ/kg. The high activation energy of the paraffin (70%)/MS1 SS-PCM verifies that the shape and thermal properties can be maintained stably during phase change conversion. The time required for SS-PCMs to complete the thermal storage and release process is reduced by 34.0% maximumly compared with that for pure paraffin, showing a lower thermal conductivity of SS-PCMs. Moreover, there is no chemical reaction between mesoporous silica and paraffin and the SS-PCMs maintain high thermal stability. In conclusion, the prepared SS-PCMs, in particular paraffin (70%)/MS1 SS-PCM, demonstrate excellent potential for application as thermal storage and insulation materials in buildings.

## Figures and Tables

**Table 1:** The mass ratios of paraffin and mesoporous silica in SS-PCMs.

Mass of Paraffin (g)	Mass of Mesoporous Silica (g)	Mass Proportion of Paraffin in SS-PCM
10.00	2.50	80%
7.50	2.50	75%
11.67	5.00	70%
9.29	5.00	65%
7.50	5.00	60%
6.11	5.00	55%

**Table 2:** Thermophysical properties of paraffin and paraffin/MS SS-PCMs.

Samples	Phase Change Temperature (°C)	*L*_test_ (kJ/kg)	*L*_theoretical_ (kJ/kg)	A (%)
Paraffin	28.5	197.4	-	-
Paraffin (70%)/MS1 SS-PCMs	23.6	135.4	138.2	98.0
Paraffin (65%)/MS2 SS-PCMs	24.2	124.9	128.3	97.3
Paraffin (60%)/MS3 SS-PCMs	25.0	116.5	118.4	98.4

**Table 3:** Activation energy calculations of paraffin/MS SS-PCMs with different pore diameters of MS.

SS-PCMs	Peak Phase Change Temperature (°C)				Activation Energy $E_{C}$ (kJ/mol)	*R* ^2^
	2 °C/min	5 °C/min	10 °C/min	15 °C/min		
Paraffin (70%)/MS1 SS-PCMs	28.02	29.27	30.37	31.27	1053.72	0.9912
Paraffin (65%)/MS2 SS-PCMs	28.97	29.72	30.77	31.77	625.38	0.9488
Paraffin (60%)/MS3 SS-PCMs	29.27	29.82	30.42	30.97	553.29	0.9722

**Table 4:** Comparison of thermophysical properties of paraffin/MS SS-PCMs with those of some SS-PCMs in literature.

SS-PCMs	Phase Change Temperature (°C)	Latent Heat (J/g)	Encapsulation Ratio (%)	References (%)
CA-PA-SA/nano SiO_2_	21.86	99.43	71.3	[16]
Paraffin/kaolin	62.4	119.49	40.0	[17]
Stearic acid/kaolinite	53.28	66.30	39%	[33]
Paraffin/palygorskite	58.55	89.2	45.5%	[38]
LA-SA/diatomite	31.17	117.30	72.2%	[39]
Paraffin/SiO_2_	26.12	111.7	64.8	[24]
Lauric acid/SiO_2_	41.5	93.8	56.6	[23]
N-heptadecane/SiO_2_	25.6	123.8	54.6%	[25]
Paraffin(70%)/MS1 SS-PCM	23.6	135.4	70%	This work
Paraffin(65%)/MS2 SS-PCM	24.2	124.9	65%	This work
Paraffin(60%)/MS3 SS-PCM	25.0	116.5	60%	This work

## Data Availability

Data is contained within the article.
